# The Effects of Disease-Induced Juvenile Mortality on the Transient and Asymptotic Population Dynamics of Chinook Salmon (*Oncorhynchus tshawytscha*)

**DOI:** 10.1371/journal.pone.0085464

**Published:** 2014-01-10

**Authors:** Masami Fujiwara, Michael S. Mohr, Aaron Greenberg

**Affiliations:** 1 Department of Wildlife and Fisheries Sciences, Texas A&M University, College Station, Texas, United States of America; 2 Fisheries Ecology Division, Southwest Fisheries Science Center, National Marine Fisheries Service, National Oceanic and Atmospheric Administration, Santa Cruz, California, United States of America; 3 Institute of Marine Sciences, University of California Santa Cruz, Santa Cruz, California, United States of America; Texas Tech University, United States of America

## Abstract

The effects of an increased disease mortality rate on the transient and asymptotic dynamics of Chinook salmon (*Oncorhynchus tshawytscha*) were investigated. Disease-induced mortality of juvenile salmon has become a serious concern in recent years. However, the overall effects of disease mortality on the asymptotic and transient dynamics of adult spawning abundance are still largely unknown. We explored various scenarios with regard to the density-dependent process, the distribution of survivorship over the juvenile phase, the disease mortality rate, and the infusion of stray hatchery fish. Our results suggest that the sensitivity to the disease mortality rate of the equilibrium adult spawning abundance and resilience (asymptotic return rate toward this equilibrium following a small perturbation) varied widely and differently depending on the scenario. The resilience and coefficient of variation of adult spawning abundance following a large perturbation were consistent with each other under the scenarios investigated. We conclude that the increase in disease mortality likely has an effect on fishery yield under a fluctuating environment, not only because the mean equilibrium adult spawning abundance has likely been reduced, but also because the resilience has likely decreased and the variance in adult spawning abundance has likely increased. We also infer the importance of incorporating finer-scale spatiotemporal information into population models and demonstrate a means for doing so within a matrix population modeling framework.

## Introduction

The effects of infectious disease on salmon populations are a serious concern. Studies suggest that Chinook salmon (*Oncorhynchus tshawytscha*) die from various infectious diseases including those caused by viruses e.g. [1], [2], bacteria e.g. [2], [3], [4], [5], [6], and metazoan parasites including myxozoans e.g. [7], [8], [9], [10] and trematodes e.g. [4], [6], [11], [12]. Recent articles [13], [14], [15] emphasize the importance of understanding the effect of infectious disease on salmon populations. However, the extent to which infectious diseases affect overall salmon population dynamics is still largely unknown because increased mortality during an early life phase may be compensated for by increased survival during a later phase [16]. The effect of the disease-induced mortality (“disease mortality”) would be nonlinear under such a compensation process. Assessing these nonlinear interactions is complicated because of the lack of information concerning the density-dependent process and the wide range of possible outcomes under the process. In addition, we also lack information on how mortality is distributed over age during the early life phases of salmon. This further complicates our understanding of the effect of infectious disease on overall population dynamics.

The objective of this study was to investigate the effects of disease mortality on the transient and asymptotic population dynamics of Chinook salmon. With respect to asymptotic dynamics, we evaluate the equilibrium adult spawning abundance, its sensitivity to disease mortality, and resilience (asymptotic rate of return toward this equilibrium abundance following a small perturbation). With respect to transient dynamics, we evaluate the deviation of the adult spawning abundance from equilibrium over time following a large perturbation. These quantities are evaluated under various scenarios with regard to the density-dependent process, the distribution of survivorship over the juvenile phase, the disease mortality rate, and the infusion of stray hatchery fish.

**Figure 1:**
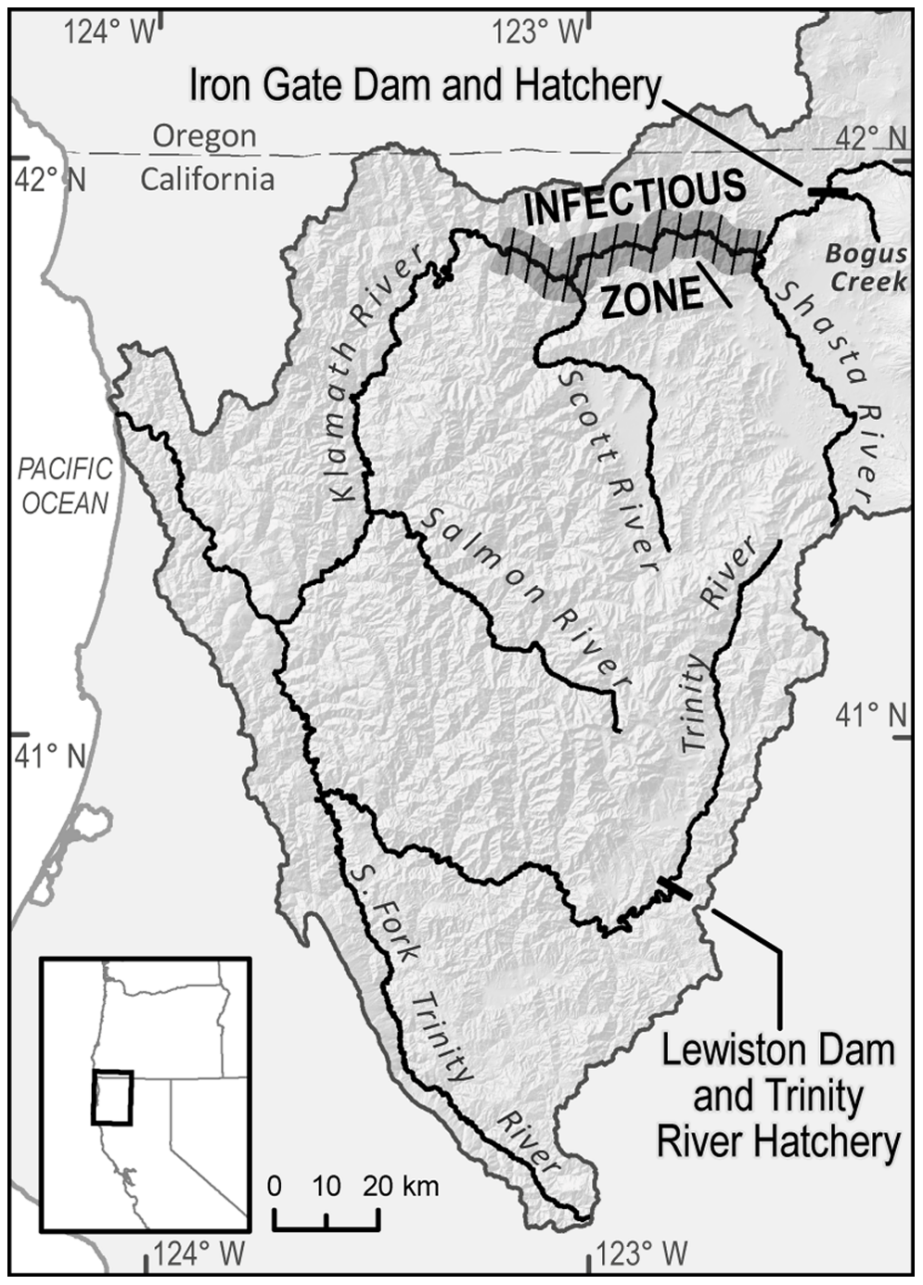
Map of the Klamath River basin.

Our study was motivated by recent reports that a large proportion of juvenile fall-run Chinook salmon out-migrants in the Klamath River (California, USA) are infected by the myxozoan parasite *Ceratomyxa shasta* [7], [9], [17], [18]. An elevated concentration of *C. shasta* has been documented in a stretch of the Klamath River main stem (Figure 1) [19], [20], [21], referred to herein as the “infectious zone”. Juvenile fall-run Chinook salmon are infected by the parasite while passing through this zone during their spring downstream migration to the ocean. Although the parasite probably has persisted in the Klamath River at a low level for a long time [22], [23], in recent years, a high proportion of the downstream migrating fish have been infected by *C. shasta*: 34% in 2004 [7], 62% in 2005 [9], 35% in 2006 [24], 31% in 2007 [18], and 49% in 2008 [25]. Once juvenile salmon are infected, they die shortly thereafter. Drawing conclusions about the interannual variation in these estimates is difficult because of the different analytical methods employed, but the high rate of infection is a serious concern. Our study focused on this particular salmon stock and parasitic disease, but the results provide insight into the effects of increased mortality during the juvenile life stage of salmon in general.

The lifecycle of *C. shasta* requires two hosts. The spores of *C. shasta* that infect juvenile salmon are released from the polychaete *Manayunkia speciosa*, the secondary host of the parasite [19]. The infectious zone has a much greater density of infected polychaetes than surrounding locations [17], [21] although it is not yet clear why this is the case. It has been suggested that the polychaetes become infected by spores released during the fall and winter from carcasses of adult salmon that were infected while passing through the infectious zone during their upstream spawning migration [17], [19].

Stage-structured population models are used to investigate the effects of an increased disease mortality rate on the transient and asymptotic dynamics of salmon populations. We employ matrix population models that are tributary-specific and include a juvenile survival rate that is dependent on the tributary's juvenile abundance. An alternative specification would be a juvenile survival rate that is dependent on the total juvenile abundance of several tributaries, or perhaps all tributaries and hatcheries. However, this specification would amount to a competition model in which groups of individuals compete for a common resource such as food or space. Under a competition model, the competitively weaker tributary populations would be eliminated, unless the strength of competition between the populations was weak [26]. Because tributary-specific populations persist in the Klamath River, we assume that inter-tributary competition of individuals is much weaker than intra-tributary competition of individuals. In addition, density-dependent matrix population models provide a convenient means by which to conduct ecosystem diagnosis and treatment (EDT) analyses [27]. Our modeling approach complements more complex approaches, such as the SHIRAZ model [28] which is suitable for simulating large-scale, basin-wide dynamics, and simpler approaches, such as logistic models, e.g. [29], which are suitable for non-species-specific, theoretical studies. The theory of matrix population models is well-developed, see [30], and this greatly facilitated our analysis.

Recently, Worden et al. [31] showed that decreased adult survival of Chinook salmon can emphasize low frequency fluctuations in population abundance. Their results showed that, depending on which part of the life history is affected by environmental fluctuation, its effect can be different. Here, we investigate how the timing of disease mortality and compensatory density dependence relative to the distribution of survivorship over the juvenile phase affects the transient and asymptotic dynamics of two Chinook salmon spawning populations, one of which receives a significant number of stray hatchery fish.

## Materials and Methods

A stage-structured matrix population model [30] with Beverton-Holt compensatory density dependence [32] was used in this study to emulate the life history of fall-run Chinook salmon. The model consists of five stages, and was applied to two spawning populations. Parameter values for the model were obtained primarily from the existing literature on Klamath River fall-run Chinook salmon.

### Life History of Klamath River Fall-Run Chinook Salmon

In the Klamath River basin, fall-run Chinook salmon spawn in the second, third, fourth, or fifth fall after having been spawned by their parents; these are termed age 2, 3, 4, and 5 spawning adults, respectively. Eggs hatch during the following winter and spring. Juveniles migrate downstream to the ocean during the spring and summer following hatching, and it is during this migration that they become infected by *C. shasta*. In addition, hatchery-produced fish are released from Iron Gate and Trinity River Hatcheries into the Klamath and Trinity Rivers, respectively, and these fish are thought to migrate to the ocean relatively quickly. However, fish released from Iron Gate Hatchery pass through the infectious zone, and they are known to be infected by *C. shasta*. On their return from the ocean some of the hatchery-produced fish “stray” into natural areas to spawn instead of returning to the hatchery. In particular, some of the fish released from Iron Gate Hatchery spawn in Bogus Creek and the upper main stem of the Klamath River below Iron Gate Dam, and some from the Trinity River Hatchery spawn in the upper main stem of the Trinity River below Lewiston Dam (Figure 1).

The approximate location of the infectious zone in the Klamath River extends from the Shasta River confluence to just below the Scott River confluence (Figure 1). Thus, the disease primarily affects juvenile salmon originating above this zone, which includes Iron Gate Hatchery, the upper Klamath River main stem, Bogus Creek, and the Shasta River. Juvenile salmon originating below this zone, e.g., in the Salmon and Trinity Rivers, are thought to be much less affected by the parasite [33]. There is some uncertainty as to whether juvenile fish from the Scott River are significantly affected by the disease. If they are, the effect is probably less than on those originating from locations further upstream because juveniles from the Scott River are exposed to a shorter length of the infectious zone during their outmigration.

We modeled the population dynamics of fall-run Chinook salmon that spawned in Bogus Creek and the Shasta River. Juvenile fish of both tributaries are affected by the disease, but the number of hatchery spawners straying into Bogus Creek (next to the hatchery) is significantly greater than the number straying into the Shasta River. The dynamics for the Klamath River upper main stem spawning population are expected to be similar to that of Bogus Creek, while the remaining natural populations of the river system are minimally affected by the disease.

### Population Model

**Figure 2:**
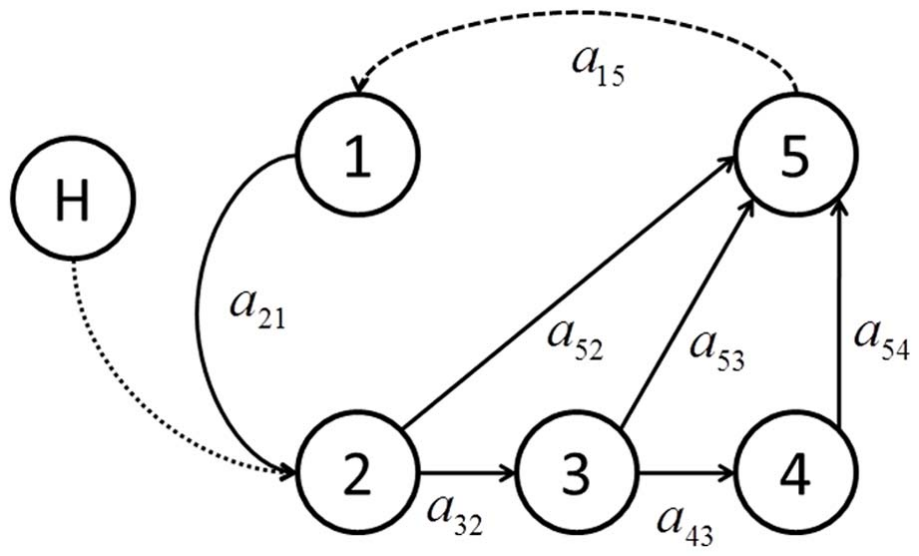
Lifecycle graph for fall-run Chinook salmon in the Klamath River basin. Circles indicate different life stages, the solid arrows indicate possible transitions of individuals, and the dashed arrows indicate fecundity. The “*a*” parameters are the matrix **A** transition rates associated with the process indicated by the arrows. See text and Table 1 for details.

A stage-structured matrix population model was used to characterize the life cycle of a single spawning population (tributary) of fall-run Chinook salmon in the Klamath River basin (Figure 2). Stage 1 represents eggs, and stage 5 represents age 3, 4, and 5 spawning adults combined. Stages 2, 3, and 4 represent subadults in the ocean that did not mature at ages 2, 3, and 4, respectively, and remain in the ocean at the outset of age 3, 4, and 5, respectively. Age 2 spawning fish were not included in the model because they are a small component of the total spawning abundance. The model projects stages from one fall to the next except for the transitions from stage 5 (spawning adult stage) to stage 1 (eggs) and the transition from stage 1 to stage 2. In the model, the spawning adult to egg stage transition was assumed to be an instantaneous event, and the eggs to stage 2 transition spanned two years. This modification in time steps is allowed because it preserves the two years necessary for individuals to transition from spawning events to stage 2, which takes two time-step projections of the model. An advantage of this approach is that a single parameter represents the survival for the first two years of life, which includes a density-dependent process and disease mortality. Furthermore, although an estimate for the survival rate from the egg stage to age 2 is available, the distribution of survivorship over those two years is unknown. A drawback of the approach is that the time associated with stage 1 is one year prior to that of the other stages. Nevertheless, the egg stage to stage 2 two year transition time is immaterial to the results of the analysis, which are expressed in terms of adult spawning abundance.

Let 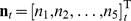, the column vector of stage-specific abundance at time *t,* with T denoting the transpose operator. Absent stray hatchery fish, the abundance at time *t*+1 is
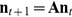
(1)with the projection matrix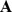 defined as
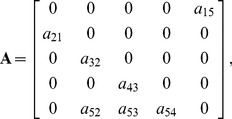
(2)where 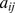 for 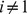 is the rate of transition of individuals from stage 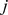 to stage 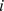 and 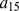 is the per-capita fecundity (Table 1). The per-capita fecundity was obtained by dividing the estimated average fecundity per female, 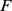, by 2, assuming an equal proportion of female and male spawners. Use of a single valued 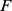 rather than an age-dependent 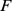 is justified given the relatively gradual increase in fecundity with size, and the considerable overlap in the distributions of size-at-age in this species [34].

**Table 1:** Population parameters for Klamath River fall-run Chinook salmon (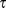 is brood year).

Parameters	Symbol	Mean	SD	Years	Source
Annual rate of surviving natural mortality from fall of 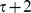	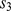	0.58	–	–	[35]
Annual rate of surviving natural mortality from fall of 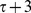 and thereafter	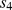	0.8	–	–	[35]
Historical^1^ survival rate from hatchery release to fall of 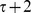	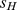	0.0087	–	1991–2004	[16] ^2^
Annual ocean fishery harvest rate from fall of 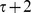	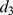	0.047	0.044	2001–2006	[36]
Annual ocean fishery harvest rate from fall of 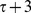 and thereafter	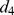	0.18	0.09	2001–2006	[36]
River fishery harvest rate during fall of 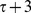	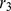	0.17	0.05	2001–2006	[36]
River fishery harvest rate during fall of 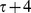 and 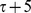	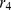	0.28	0.11	2001–2006	[36]
Maturation rate during fall of 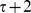	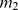	0.05	0.08	1979–2003	[36]
Maturation rate during fall of 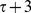	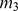	0.38	0.12	1979–2003	[36]
Maturation rate during fall of 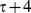	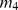	0.87	0.10	1979–2003	[36]
Fecundity for age 3 ( 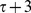 ), age 4 ( 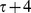 ), and age 5 ( 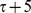 )	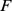	3634	–	–	[37]
Number of fish (fingerling) released from Iron Gate Hatchery	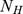	5,100,000	–	–	[38]
Hatchery fish stray rate in Bogus Creek	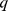	0.11	0.05	2007–2010	See text
Hatchery fish stray rate in Shasta River	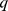	0	–	–	
Historical^1^ equilibrium survival rate from egg to fall of 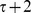 in Bogus Creek	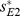	0.0014617	–	–	See text
Historical^1^ equilibrium survival rate from egg to fall of 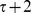 in Shasta River	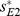	0.0017613	–	–	See text
Projection matrix 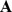 transition rates (Equation 2)					
	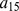	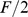	–	–	
	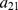	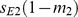	–	–	Equation (5)
	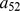	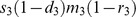	–	–	
	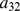	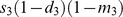	–	–	
	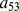	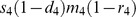	–	–	
	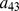	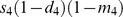	–	–	
	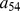	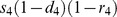	–	–	
Hatchery fish stray contribution, stage 2	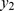	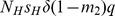	–	–	

1. Pre-elevated infectious disease.

2. Geometric mean of estimates.

Stray hatchery-origin fish contributing to a natural spawning population were combined with the natural-origin fish in the model at stage 2 (Figure 2). The straying of these fish does not actually occur until they reach the natural spawning area as adults, but because these fish are believed to be governed by the same transition rates for stages 2–5 [35], it was mathematically more convenient for us to merge these two components at the stage 2 node. With the stray hatchery fish, the abundance at time *t*+1 is
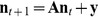
(3)with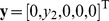, where 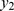 is the contribution of stray hatchery fish at stage 2 (Table 1).

The effects of disease mortality on natural production and the contribution of stray hatchery fish are incorporated into the model as described in the next section.

### Disease Submodels

**Figure 3:**
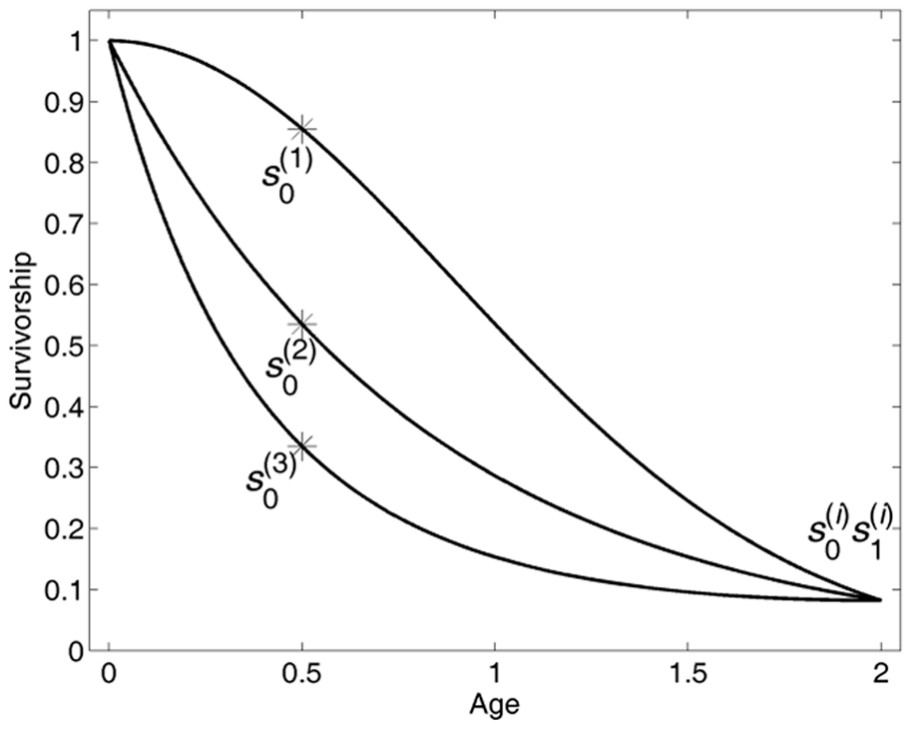
Three hypothetical density-independent survivorship curves from the egg stage to stage 2 (first two years of life) with (1) increasing, (2) constant, and (3) decreasing instantaneous mortality rates. Stars indicate the survivorship through age 0.5 under these three scenarios. The height of the star is equal to the survival rate through age 0.5 (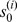) under scenario *i*, and the height of the end point at age 2 is the survival rate over two years (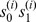), which is the same under each of the three scenarios in this example.

It is generally thought that cohort strength in salmon is established by the end of early ocean life [39] and, for Klamath River fall-run Chinook, that this occurs prior to stage 2 [35]. However, there is substantial uncertainty about the shape of the survivorship curve for juveniles between the egg stage and stage 2, and about when and where compensatory density dependence takes place. This has implications for the impact of disease mortality on a population. Consider for example the three hypothetical juvenile density-independent survivorship curves for the first two years of life displayed in Figure 3. The three curves represent scenarios in which the instantaneous mortality rate is linearly increasing, constant, and linearly decreasing with age (top to bottom) although the overall two-year mortality rate is identical. If disease mortality occurs when individuals are age 0.5, then the number of individuals alive just prior to the disease event varies substantially among the three scenarios, even though the survival rate over the 2 year period is the same. Also the density-dependent compensatory processes may affect the survival before or after the disease mortality. Thus, the effect of the compensatory process can vary substantially depending on its timing and the distribution of survivorship over age. The spawner-recruit component of the typical matrix population model ignores this level of detail.

The distribution of survivorship over the juvenile phase and the timing of disease infection were incorporated into the 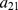 transition rate (Equation 2, Table 1) as follows. The survival rate of naturally-produced fish during this transition from the egg stage to stage 2, 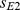, was formulated with density-dependent survival occurring either before the infectious zone (Model DD-pre), or after the infectious zone (Model DD-post), but not both. The Beverton-Holt [32] form of density-dependent survival was assumed, with a maximum possible value of 1. For the density-independent component of survival, it was assumed that disease infection/mortality is an instantaneous event, and that the disease mortality rate is density independent. Thus, for Model DD-pre, density-dependent survival 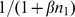 before the infectious zone, where 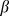 is the parameter for density dependence, is followed by density-independent survival 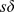 to stage 2, where 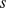 is the density-independent historical (pre-elevated infectious disease) survival rate, and 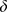 is the density-independent current (post-elevated infectious disease) survival rate relative to that of the historical period, so that 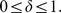 For Model DD-post, density-independent survival 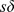 through the infectious zone is followed by density-dependent survival 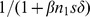 to stage 2 and thus
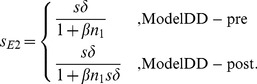
(4)


We chose to re-parameterize 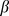 in terms of the historical equilibrium values 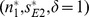 by substituting these quantities into Equation (4) and solving for 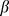. Substituting these respective values of 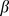 back into Equation (4) yields
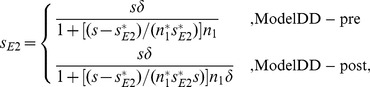
(5)with 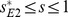.

The survival rate of hatchery-produced fish from release to stage 2, on which the contribution of stray hatchery fish, 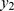, depends (Table 1), was assumed to be density independent, 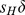, where 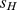 denotes the historical (pre-elevated infectious disease) survival rate over this life-history phase of hatchery released fish. Density independent survival was assumed for hatchery-released fish because the number of fish released from the hatcheries varies little from year to year, and because these fish are thought to migrate to the ocean relatively quickly. However, the offspring of stray hatchery fish do experience density dependent survival between stage 1 and stage 2 during their rearing in the natural environment.

Our disease submodels consider two simple alternatives with regard to the timing of the density-dependent compensatory process relative to the disease mortality. However, there are other possibilities. For example, density-independent survival could occur both before and after the density-dependent process. Or, the disease mortality could occur during the density-dependent process, resulting in the density-dependent term being partitioned (i.e., introducing before and after disease mortality terms). Although these alternatives may more closely represent the natural course of events, we believe that our simpler models provide insights into these more complex situations without the complications introduced by the additional model structure (see *Discussion*).

### Parameter Values

The life history parameters governing the projection matrix 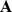 transition rates and the stray hatchery fish contribution 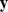 were assumed to be the same for the Bogus Creek and Shasta River populations, except for 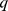, 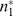 and 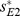. Values for the in-common life history parameters, including fecundity, age-specific survival, harvest, and maturation rates, and the number of hatchery fish released and their historical survival rate to age 2, were obtained from the existing literature on Klamath River fall-run Chinook salmon (Table 1). Estimates for 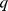, 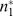 and 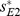 were obtained separately for the two populations as described below. The remaining parameters, 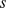 and 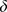, were treated as unknown variables in the analysis.

With respect to estimation of the stray rate, 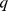, many of the fish produced at Iron Gate Hatchery receive coded-wire tags, and this is done in a manner that facilitates expansion of observed adult tag recoveries in a particular tributary to an estimate of the total number of hatchery-origin fish present in that tributary. The fraction of returning hatchery-origin adult fish that stray into Bogus Creek for spawning (versus return to the hatchery or to other tributaries) is the stray rate, and the mean estimated value of this quantity was 0.11 (Table 1). The stray rate of hatchery-origin fish into the Shasta River is very low (<0.01), and for the purposes of this study, it was assumed to be equal to zero.

To estimate 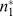 and 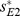, we assumed that the two populations had been in equilibrium. That is, we set the stage 5 abundance at equilibrium, 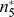, equal to the average historical abundance of spawning adults for the respective population (Table 2), from which 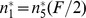. We then estimated 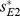 as that value, which when coupled with the Table 1 values, 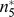, and 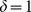, yielded an annual population growth rate of 1 for the Shasta River (Equation 1) and Bogus Creek (Equation 3) populations, respectively. The estimated 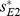 was 0.0017613 for the Shasta River, and 0.0014617 for Bogus Creek.

**Table 2:** Spawning abundance (females and males) of two natural area populations and Iron Gate hatchery (2001–2006).

Location	Mean	SD	Source
Bogus Creek	9446	6193	[40]
Shasta River	3776	3153	[40]
Iron Gate Hatchery	21497	11269	[40]

### Analyses

For the analysis we varied the unknown density-independent survival components, 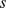 and 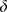, of Models DD-pre and DD-post over their respective range, 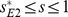 and 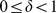, to evaluate their individual effects on the spawning population asymptotic and transient dynamics. By varying the 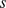 component of these models, we were investigating the effect of a varying distribution of survivorship over the first two years of life (see Figure 3), unrelated to the increase in disease mortality. The specific aspects of the asymptotic and transient dynamics that were evaluated included the equilibrium adult spawning abundance level, its sensitivity to disease mortality, its resilience, and the deviation in the adult spawning abundance from equilibrium over time following a large perturbation, as described below.

Because the model includes compensatory density dependent survival, it has a stable equilibrium point which is independent of the initial condition. Thus, the equilibrium adult spawning abundance was determined by projecting an arbitrary positive population abundance vector over 1000 time steps using Equation (1) or (3), as appropriate, and comparing the vectors at the final two time steps to confirm convergence to the stable equilibrium point.

The sensitivity of the equilibrium abundance to disease mortality was defined as the derivative of the equilibrium abundance, 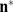, with respect to the disease survival parameter, 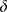. For the model including stray hatchery fish (Equation 3) this was calculated as (see Equation 70 in [41]),
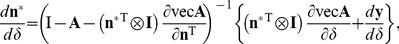
(6)where 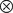 denotes the Kronecker product operator, 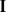 is a 5×5 identity matrix, 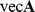 is a 25×1 vector in which the columns of 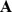 are stacked sequentially below one another, 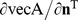 is a 25×5 matrix where element 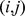 is the partial derivative of 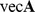 element 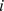 with respect to 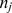, 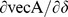 is a 25×1 vector where element 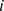 is the partial derivative of 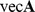 element 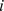 with respect to 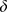, 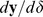 is a 5×1 vector where element 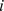 is the derivative of 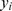 with respect to 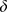, and all quantities were evaluated at 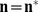. Equation (6) was also used to calculate the sensitivity of the equilibrium abundance to disease mortality for the model not including stray hatchery fish (Equation 1), by substituting 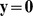 into the last term of Equation (6).

Resilience has been defined as the asymptotic rate of return to equilibrium abundance (asymptotic rate of decay in the magnitude of the deviations 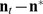) following a small perturbation [42], [43]. For discrete-time, discrete-stage, nonlinear population models in the form of equation (1), resilience is measured as 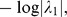 where 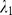 is the dominant eigenvalue of the Jacobean evaluated at the equilibrium point. We used this metric of resilience, calculating the Jacobean as (see Equation 20 in [44]),
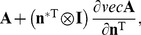
(7)with all quantities evaluated at 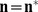. We thus examined the resilience of the Shasta River population as a function of the two density-independent survival components, 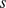 and 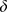, for the DD-pre and DD-post models. Equation (7) is not directly applicable to the Bogus Creek population because of the stray hatchery fish contribution term in equation (3).

To compare the transient dynamics of the two populations, we repeatedly simulated a large, random perturbation away from the equilibrium abundance and followed the deviation decays in adult spawning abundance over time under selected parameter values. Large perturbations from equilibrium are consistent with the variability in adult spawning abundance observed in these populations (Table 2). The evaluation was conducted as follows. From their equilibrium at 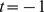, the populations were randomly perturbed at 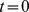 (described below), and then projected into the future 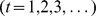 using Equations (1) or (3), as appropriate. This sequence was repeated 500 times and the coefficient of variation (CV) of the adult spawning abundance (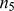) at each time step, 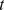, was calculated. Models DD-pre and DD-post were thus evaluated for each population with 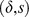 values of (0.1,0.1) and (0.9,0.9) that were previously found (see Results) to result in relatively low and high resilience, respectively, in the Shasta River population. The random perturbation above was introduced in such a way that the total reproductive value of the population before and immediately after the perturbation was equal. This was done by calculating the perturbed 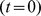 stage 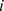 abundance as
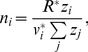
(8)where 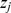 is a random number uniformly distributed between 0 and 1, and the total reproductive value at equilibrium, 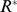, was
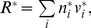
(9)where 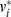 is the reproductive value of stage 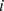 at equilibrium [30]. This ensured that the magnitude (Euclidean norm) of the perturbation was invariant among simulations for a given population, model, and resilience level. This was important because if the magnitude of the perturbation was allowed to vary it would confound the comparison of the results among populations, models, and resilience levels. Instead, the population abundance vector was perturbed in different directions by the same amount.

### Ethics Statement

No new data was collected for this study, and ethical approval for this study was not required. All of the data are available from the sources cited.

## Results

**Figure 4:**
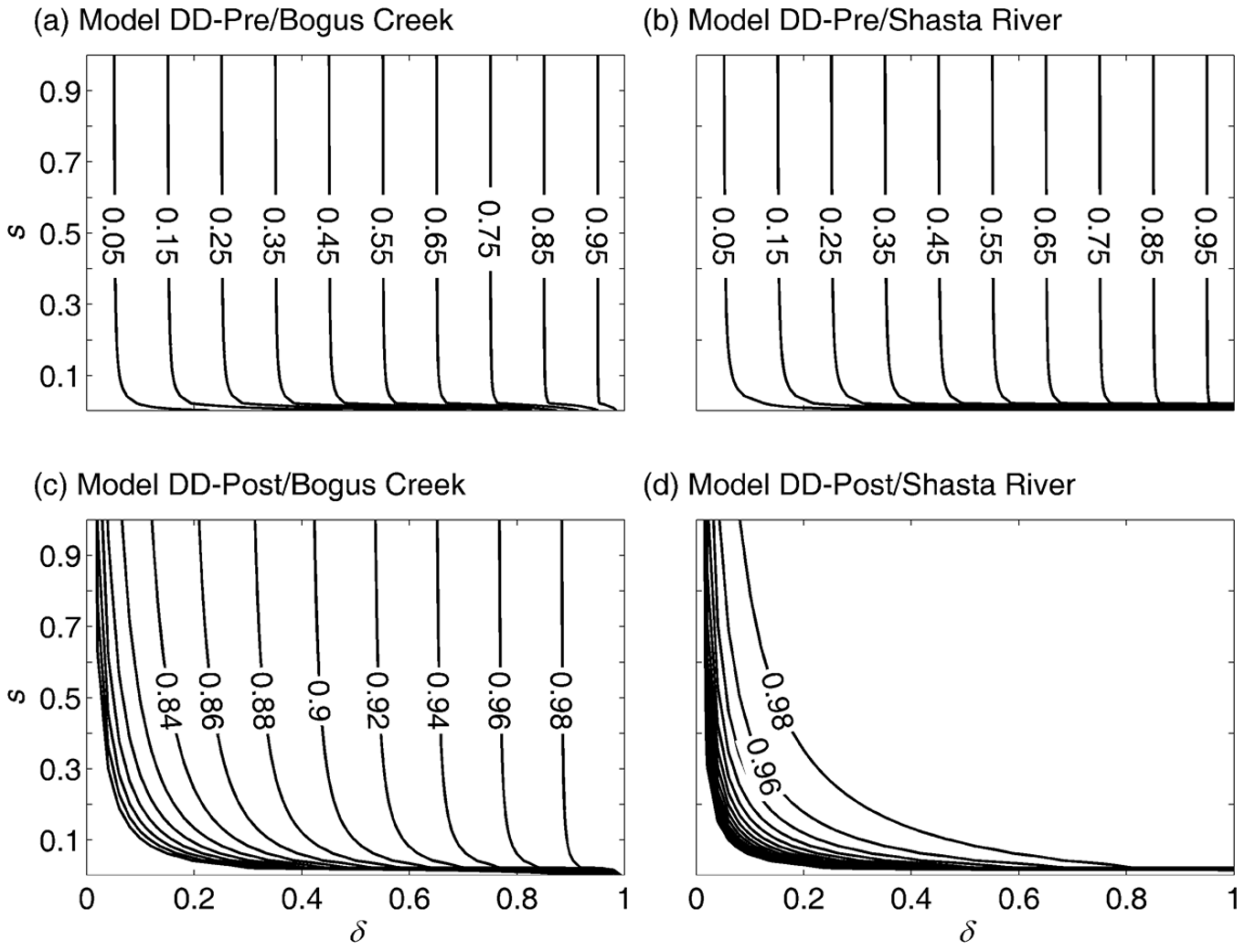
Equilibrium adult spawning abundance (contours) relative to that with no elevated disease 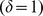 and its dependence on the density-independent survival components 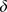 and 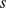, the timing of density dependence (Models DD-pre and DD-post), and the spawning population (Bogus Creek or Shasta River).

The equilibrium adult spawning abundance under recent historical conditions (no elevated disease, 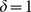) was estimated to be 9446 individuals for Bogus Creek, and 3776 individuals for the Shasta River (Table 2). The proportion of the equilibrium adult spawning abundance under current postulated conditions relative to this historical level for Models DD-pre and DD-post is shown in Figure 4. The equilibrium adult spawning abundance declined almost linearly with increases in the disease mortality rate (reduced 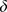) under Model DD-pre (Figure 4a and 4b). Although the effect is small, the density-dependent process in the next generation partially compensated for the loss. This is evident from the curved contours in the lower left corners of Figure 4a and 4b, where the density-dependent survival rate was already so high (because the density-independent survival was so low) that it could not be increased much further in response to the increase in disease mortality (i.e., there was little compensation). Under Model DD-post, increased disease mortality tended to be more highly compensated for by the density dependence, where the equilibrium abundance was substantially higher than in Model DD-pre given the same value of 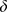 (Figure 4c and 4d). However, even for Model DD-post, when 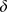 was very low, the reduced abundance could no longer be fully compensated for by the density dependence, an effect that was magnified as the survival prior to density dependence was reduced.

The sensitivity of the equilibrium adult spawning abundance 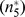 to disease mortality, and its dependence on population, density dependence timing, and resilience level is shown in Table 3. The sensitivity of the Bogus Creek population to disease mortality was at least twice that of the Shasta River population, regardless of the timing of density dependence and the resilience level. This was due to the fact that 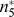 itself is greater for Bogus Creek, which includes hatchery strays, than for the Shasta River. The results also show that for Model DD-pre, within a population, the sensitivity is independent of the resilience level and its magnitude is intermediate to the values exhibited for Model DD-post. For the DD-post model, within a population, the sensitivity was negatively associated with the resilience level. Overall, the sensitivity was highest under the system with the greater equilibrium adult spawning abundance, density-dependent survival occurring after the infectious zone, and low resilience.

**Table 3:** Sensitivity (×10^4^) of Shasta River and Bogus Creek equilibrium adult spawning abundance to the disease mortality rate under low and high resilience (R) scenarios for Models DD-pre and DD-post.

	DD-Pre		DD-Post	
Location	R low	R high	R low	R high
Shasta River	0.38	0.38	0.88	0.023
Bogus Creek	0.95	0.95	2.0	0.21

**Figure 5:**
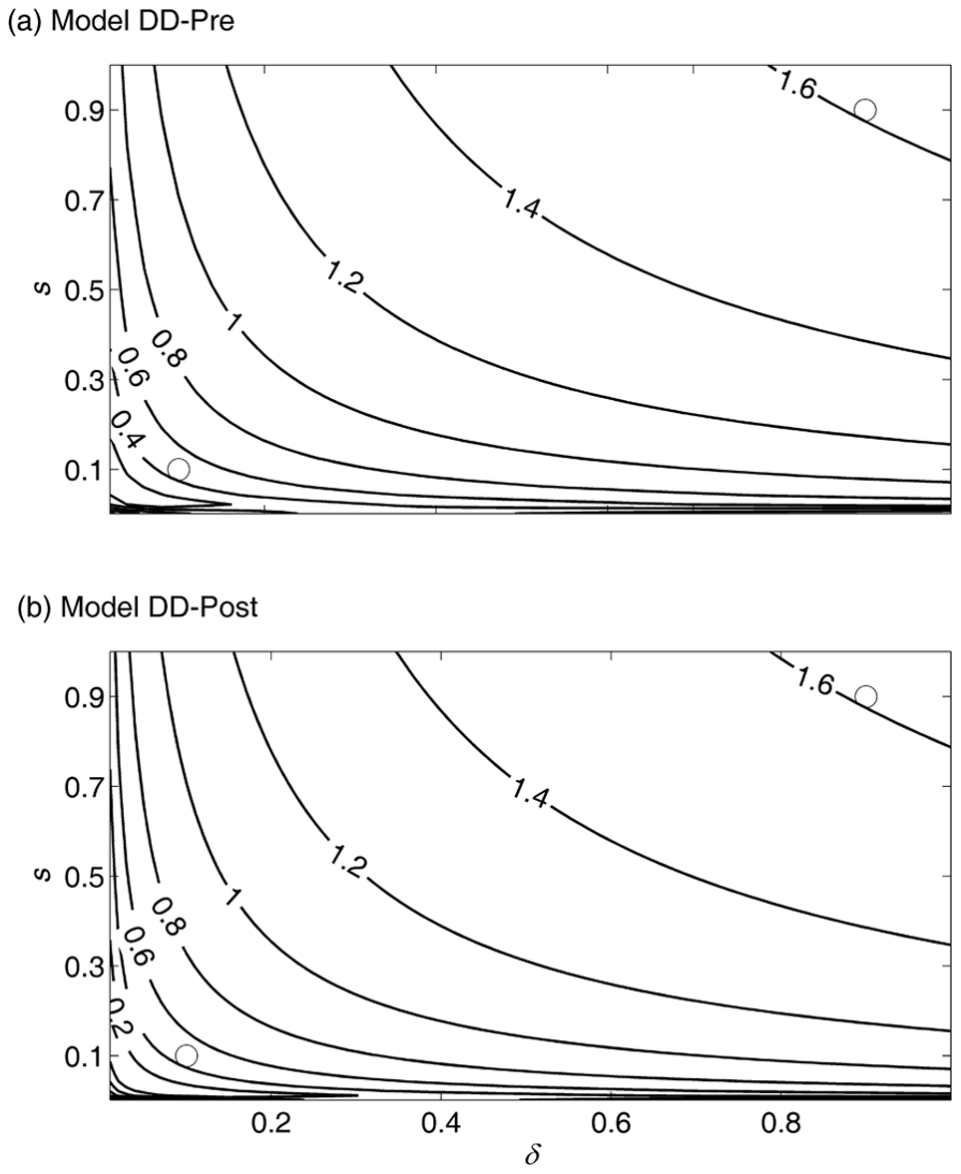
Resilience (contours) of the Shasta River adult spawning abundance and its dependence on the density-independent survival components 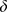 and 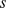, and the timing of density dependence (Models DD-pre and DD-post). Open circles indicate survival rate combinations used to generate Figure 6 results: low resilience 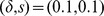; high resilience 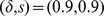.

Resilience label (low,high) denotes survival rate combinations 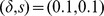 and 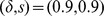, respectively, used to generate results (see Figure 5).

The resilience of the Shasta River population was very similar under the DD-pre and DD-post models (Figure 5a and 5b). Resilience decreased with increases in the disease mortality rate (reduced δ), and with decreases in the other density-independent survival component, *s*.

**Figure 6:**
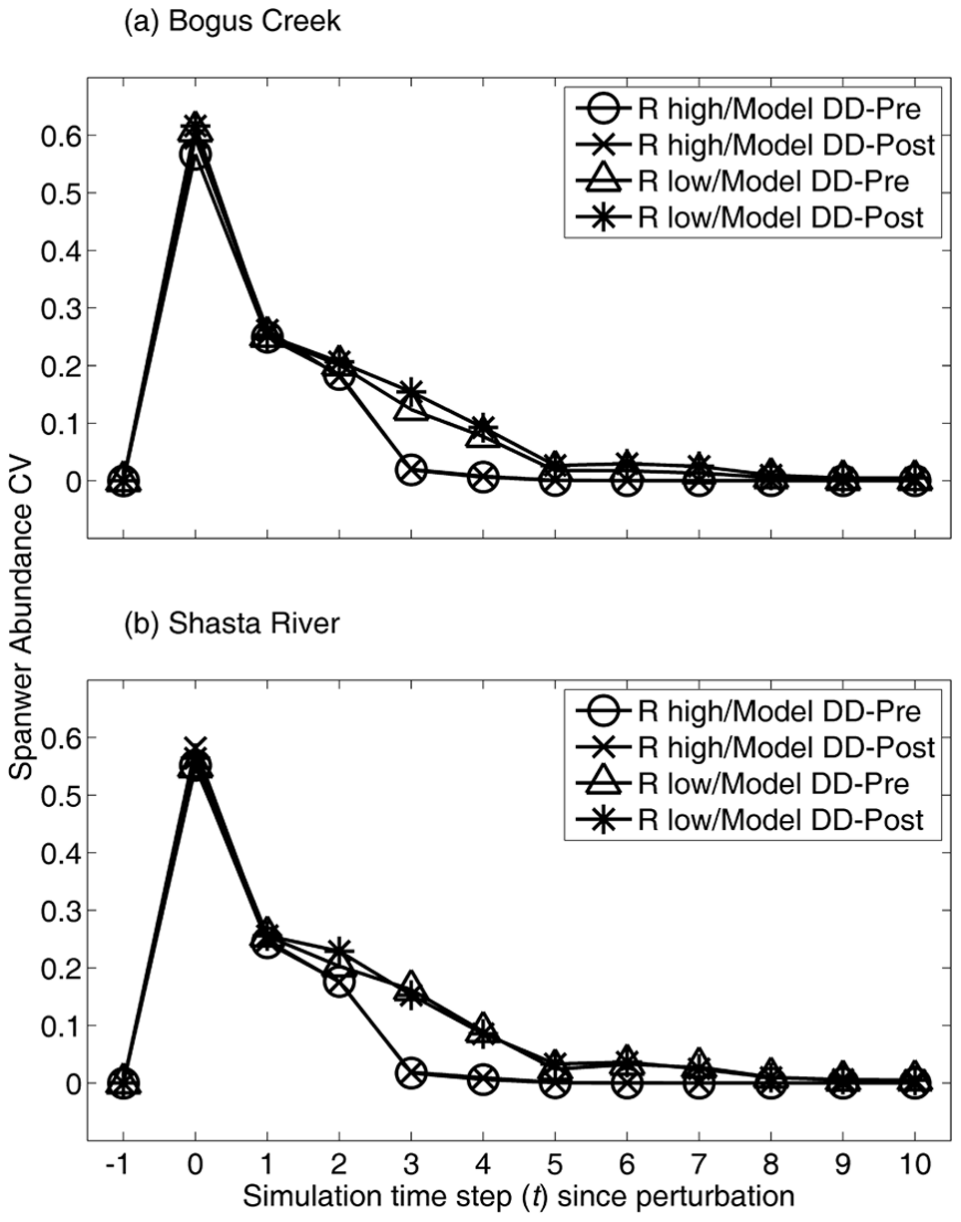
Adult spawning abundance coefficient of variation (CV) across simulations and its dependence on time step following a large perturbation (*t* = 0), resilience level (low or high), and the timing of density dependence (Model DD-pre or DD-post): (a) Bogus Creek and (b) Shasta River. Resilience label (low,high) denotes survival rate combinations 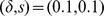 and 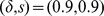, respectively, used to generate results (see Figure 5).

With respect to the transient dynamics, although the coefficient of variation in adult spawning abundance for the Shasta River and Bogus Creek was reduced at 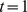 and 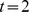 (one and two time steps following perturbation), regardless of the resilience level (Figure 6), this was not due to the compensatory process because it takes at least two time steps for the compensation effect to be realized in the adult spawning abundance. In the high-resilience system, the coefficient of variation declined quickly thereafter, but in the low-resilience system it remained at a higher level, which is consistent with the resilience results for the Shasta River population.

## Discussion

The effect of an increased disease mortality rate on the overall population dynamics of salmon is difficult to assess. This is partly due to the fact that the timing of compensatory density dependence relative to the distribution of survivorship over the juvenile phase is typically unknown. Hatchery-produced returning spawners also stray into some natural spawning populations, obscuring the effect of such increased mortality. The complex life history of salmon further complicates the dynamics [28]. To gain insight into the effect of an increased disease mortality rate on the overall population dynamics of salmon, we formulated a five-stage population model for Chinook salmon and used it to explore a range of plausible scenarios regarding their early life survival, including an increase in the disease mortality rate.

Spawning abundance is affected by an increased disease mortality rate, but the effect depends on the timing of the density-dependent compensatory process (Figure 4). If this process occurs before the increased mortality, then the effect on the equilibrium adult spawning abundance is nearly a linearly decreasing function of the mortality rate (Figure 4a and 4b). However, if the process takes place after the increased mortality, the effect may be significantly reduced (Figure 4c and 4d). Thus, absent knowledge of when the density dependence process occurs, it is only possible to describe broad ranges for the effect.

How the survivorship of a cohort is distributed over the juvenile phase also influences the magnitude of the effect of an increased disease mortality rate. If the density dependence acts during a time when the density-dependent survival rate is already high, it cannot fully compensate for the increased mortality (lower left corner of Figure 4a and 4b). This is true even if the density dependence is acting after the increased mortality (lower left corner of Figure 4c and 4d). Thus, to assess the impact of a disease mortality rate increase on the population dynamics, one must also have an estimate of the distribution of survivorship over the juvenile phase. Ralston and O'Farrell [45] investigated a similar effect resulting from heterogeneously distributed fishing mortality on yield. They demonstrated the importance of incorporating the distribution of survivorship over age into a population model.

Previous studies in the Klamath River have indicated that the mortality rate of juveniles passing through the infectious zone is high [7], [9], [18], but the magnitude of this mortality rate and whether the infectious zone is a short-lived or permanent phenomenon remains uncertain. For example, a field experimental study by Ray et al. [46] demonstrated that the disease-induced mortality rate can range from 2.5% to 98.5% depending on the season, duration of exposure, and density of actinospores in the water. If disease mortality is greatly increased by passage through the infectious zone under natural conditions, and the infectious zone persists into the future, equilibrium adult spawning abundance of exposed populations may be substantially reduced if abundance is constrained by density-dependent survival before the fish reach the infectious zone (Figure 4a and 4b), but may be much less affected by increased disease mortality if subsequent density dependence allows for a strong compensatory response (Figure 4c and 4d). Therefore, estimates of the disease mortality rate alone will not allow us to understand its effect on the equilibrium adult spawning abundance or fishery yield.

However, the increased disease mortality rate is expected to reduce the resilience of the population unaffected by hatchery fish (Figure 5), and will lengthen the time necessary to recover from future pulse changes in the mortality schedule (Figure 6b). Thus, if the increased disease mortality rate is permanent, the population is expected to maintain a greater deviation from the equilibrium adult spawning abundance, at least in the short term, following a natural perturbation (e.g., drought or high stream temperature) or anthropogenic perturbation (e.g., water withdrawal or increased fishing pressure). Because such perturbations are a regular occurrence, the loss of resilience is expected to result in the prolonged suppression of adult spawning abundance over time. This finding complements that of Worden et al. [31] who showed that increased mortality of adults can emphasize low frequency fluctuation in adult spawning abundance, which effectively reduces the resilience of the population. We thus conclude that the increase in disease mortality likely has an effect on fishery yield under a fluctuating environment, not only because the mean equilibrium adult spawning abundance has likely been reduced, but also because the variance in adult spawning abundance has likely increased.

The sensitivity of the equilibrium adult spawning abundance to the disease mortality rate also differs substantially among different scenarios that we investigated. With the DD-post model, reduced resilience was associated with increased sensitivity, whereas with Model DD-pre the sensitivity was unassociated with the resilience level. This too suggests the importance of measuring the distribution of survivorship and the relative location/timing of density dependence to more fully understand the effect of disease mortality on the population dynamics.

Hatchery production and the straying of hatchery-origin spawners into natural spawning areas are other factors that can complicate our understanding of natural spawning population dynamics. In our study system, Bogus Creek receives stray hatchery fish whereas the Shasta River does not. Under Model DD-post, the Bogus Creek equilibrium adult spawning abundance declines faster with increases in the disease mortality rate than it does in the Shasta River (Figure 4c versus 4d). This results from the assumption that hatchery-origin fish do not experience compensatory density dependence in their first two years of life, and thus the Shasta River population exhibits stronger compensation than does the Bogus Creek population. On the other hand, the coefficient of variation in adult spawning abundance following a large perturbation appears to be unaffected by the infusion of stray hatchery fish (Figure 6). This was counter-intuitive because stronger compensation was expected to lead to more quickly damped deviations in spawning abundance. Also, hatchery fish released at the fingerling life stage were incorporated into our models, but fish are also released from the basin's hatcheries later in life at the sub-yearling stage, undoubtedly increasing the complexity of the system dynamics and the uncertainty of our understanding.

In our models, the compensatory process acts only on the naturally spawned juvenile abundance. However, it is also possible that additional density dependence is operating on the aggregate abundance of juvenile out-migrants (including hatchery production) along with the tributary-specific density dependence. If there are two density-dependent processes acting on salmon populations, one tributary-specific and the other affecting aggregate juvenile abundance, the former is likely to have the greater effect. Otherwise, one or more of the component populations would likely be eliminated because of the aggregate competition [26]. Of course, these are only some of the possibilities with respect to density-dependent juvenile mortality. This suggests the need for further investigations of the dynamics of populations experiencing multiple density dependencies and stock mitigation/supplementation.

Density dependencies that are more complex than those explored in this study may occur in nature. For example, the density dependence may be operating during a shorter period of time. If so, this would effectively add density-independent survival terms before and after the density-dependent term in our model. Additional density-independent survival would be expected to simply increase the survival rate that the density dependence is acting upon. Alternatively, the disease mortality may act in the middle of the compensatory density-dependent process, splitting the effect in two (before and after disease mortality). This could be accommodated by combining our two density-dependent survival terms in the five-stage model. With this combination, it would be likely that an increased disease mortality rate would reduce the resilience of the system and also would reduce the equilibrium adult spawning abundance. However, the magnitude of the reduction would depend on how the reduced survival was distributed among the two density-dependent terms and on the density-independent survival rate.

Along with obtaining a better understanding of the distribution of survivorship over the juvenile phase and the timing of density-dependent mortality, relating the infection processes of *C. shasta* to the disease-induced mortality of salmon is a key next step towards developing a predictive salmon/disease population dynamics model. The lack of historical monitoring of the disease-causing agents (e.g., the density of *C. shasta* actinospores or *M. speciosa* polychaetes in the infectious zone) will likely prevent construction of a statistically-based model [16] for this purpose. However, it appears that a submodel for the infection process could be developed based on the differential equation model provided by Ray et al. [46], parameterized with experimentally-derived estimates, and incorporated into the stage-structured salmon population model presented in this paper. Such a model could be very useful for predicting the effect of ceratomyxosis, provided the density of actinospores and/or polychaetes in the infectious zone is regularly monitored going forward.

An important message from our study is that simple population models such as standard spawner-recruitment models and standard matrix population models alone are not fully adequate to assess the impact of an increased disease mortality rate at a particular time within a stage because of the time-scale mismatch between these models and the process. It is important to know how survival is distributed throughout the stage within which the increased mortality rate acts, and potentially the location within a spatially stratified population or meta-population in which it acts. The effects of an increased disease mortality rate may also be countered to some degree by the infusion of stray hatchery fish to a natural spawning population. This coupled with the likely expression of density-dependent effects at the local level suggests that, for some situations, the modeling of salmon population dynamics is likely more appropriate at the tributary scale than at the basin-wide scale.
